# Investigating the role of organizational stress in nurses' psychosomatic complaints: Evidence from a study in northeastern Italy

**DOI:** 10.3934/publichealth.2024021

**Published:** 2024-03-27

**Authors:** Francesco Marcatto, Emilia Patriarca, Davide Bramuzzo, Emanuela Lucci, Francesca Larese Filon

**Affiliations:** 1 Department of Life Sciences, University of Trieste, Trieste, Italy; 2 Clinical Unit of Occupational Medicine, University of Trieste, Trieste, Italy

**Keywords:** organizational stress, nurses, psychosomatic complaints, management standards, work-related stress

## Abstract

**Background:**

Nursing is pivotal to healthcare delivery but is often associated with high levels of organizational stress. In this cross-sectional study, we aimed to investigate the associations between exposure to organizational stressors, measured using the Health and Safety Executive Management Standards Indicator Tool, and psychosomatic complaints among nurses in a medium-sized city hospital in northeastern Italy.

**Methods:**

A total of 215 nurses participated in the study, completing self-report questionnaires assessing organizational stressors and the prevalence of psychosomatic complaints experienced over the preceding six months.

**Results:**

Significant associations were observed between various organizational stressors and psychosomatic complaints among nurses. Specifically, the Relationships factor emerged as a significant predictor of palpitations, irritability, anxiety, physical and mental tiredness, and headache. Additionally, Demands and Managers' support were identified as significant predictors of specific psychosomatic complaints.

**Conclusion:**

This study highlights the critical role of addressing organizational stressors, particularly those related to interpersonal relationships, in promoting nurse well-being and optimizing patient care delivery. Despite its strengths, including the use of a well-established measurement tool and a comprehensive assessment of psychosomatic complaints, limitations such as the cross-sectional design and self-report measures warrant consideration. By prioritizing supportive work environments and implementing targeted interventions, healthcare organizations can cultivate a culture of well-being among nurses, ultimately enhancing the quality and safety of healthcare delivery.

## 1. Introduction

Nursing, which is known for its indispensable role in healthcare, is a profession that comes with multifaceted challenges. The intricate nature of healthcare settings, characterized by high workloads, time pressures, and emotional demands, predisposes nurses to elevated levels of stress [1],[2]. Occupational stress among nurses can have profound consequences for both nurses' well-being and patient care, as well as organizational effectiveness. One notable consequence of occupational stress is its detrimental effect on nurses' physical and mental health. Prolonged exposure to high levels of stress can lead to symptoms of burnout, including emotional exhaustion, depersonalization, and a reduced sense of personal accomplishment [3],[4]. This can result in increased absenteeism, turnover rates, and decreased job satisfaction among nurses, ultimately undermining their ability to perform their duties effectively and compromising the quality of care provided to patients [5]–[8]. Additionally, stress-related health issues, such as cardiovascular disease, musculoskeletal disorders, and mental health disorders like anxiety and depression, can further impair nurses' ability to fulfill their professional responsibilities [9]–[12]. Thus, the consequences of stress in nurses not only affect their own well-being but also have far-reaching implications for the quality and safety of healthcare delivery [13],[14].

Within the realm of nursing, stressors can be categorized into two broad distinct domains: Operational (also called content-factors) and organizational (also called context-factors) [15],[16]. Operational stressors encompass the immediate challenges and demands inherent in nursing practice, including high patient acuity, shift work, exposure to traumatic events, and dealing with patient suffering and death [16],[17]. In contrast, organizational stressors transcend individual tasks and encompass aspects of the work environment and organizational culture. These stressors arise from factors such as inadequate staffing levels, lack of control over one's tasks, unclear job roles, and the presence of conflict with colleagues [15].

Unlike operational stressors, which are inherent to the nature of nursing practice, organizational stressors represent a modifiable aspect of the work environment, offering opportunities for intervention and mitigation by healthcare organizations. Moreover, while both operational and organizational stressors exert significant impacts on nurses' well-being and job satisfaction, numerous studies showed that nurses frequently cite organizational factors as the main sources of stress [14]–[19]. Addressing organizational stressors is therefore essential for promoting both nurse well-being and optimal patient care outcomes.

### 1.1. The management standards framework

One notable framework that has gained prominence in addressing organizational stressors is the Health and Safety Executive (HSE) Management Standards approach [20]. Developed by the HSE in the United Kingdom, this approach provides a comprehensive framework for managing work-related stress by identifying seven key organizational stressors and implementing targeted interventions. According to this approach, the seven primary areas of work design that are crucial for promoting employee well-being and preventing stress are the following: Demands, Control, Managers' support, Peer support, Relationships, Role, and Change. The Demands area pertains to the extent and nature of the workload, including issues such as workload volume, pace of work, and the cognitive and emotional demands placed on employees. Control refers to the degree of autonomy and decision-making authority employees have over their work tasks and how much input they have in decision-making processes. Support involves the provision of adequate resources, encouragement, and assistance from supervisors (Managers' support) and colleagues (Peer support), to enable employees to carry out their work effectively. The Relationships dimension encompasses the quality of relationships within the workplace, as well as the presence of conflict or bullying. Role concerns clarity and understanding of job roles and responsibilities, and the presence/absence of conflicting roles. Last, change addresses the extent to which organizational changes are managed effectively, including communication, consultation, and employee involvement in the change process.

By systematically assessing these organizational areas, organizations can develop targeted interventions to address specific stressors and promote employee well-being. For this purpose, the HSE has developed the HSE-Management Standards Indicator Tool (HSE-MS IT) questionnaire [21]. Several studies have already demonstrated its robust psychometric properties [22], and how each scale is sensitive to different psychological and physical outcomes, including job satisfaction [23],[24], perceived stress at work [25], job-related anxiety and depression, musculoskeletal pain, hypertension and gastrointestinal disorders [26],[27], and work ability [28].

By adopting the HSE Management Standards approach, healthcare organizations can proactively manage organizational stressors within the nursing profession, thereby promoting a healthier work environment and supporting nurses in delivering high-quality care while safeguarding their own well-being [29],[30].

### 1.2. The present study

In this study, we aim to explore the associations between exposure to organizational stressors, as measured by HSE-MS IT, and a spectrum of psychosomatic complaints among nurses working in a hospital in mid-sized city in Italy. Psychosomatic complaints encompass a diverse array of physical symptoms influenced by psychological factors [31],[32], and are known to be strongly associated with work-related stress [33].

This way, we aim to shed light on the pathways through which work-related stress influences nurses' well-being. Identifying which specific organizational stressors play a significant role in the prevalence of psychosomatic complaints holds profound implications for the formulation and implementation of targeted stress management interventions, thereby bolstering nurses' well-being and improving organizational effectiveness.

## 2. Materials and methods

### 2.1. Participants and procedure

For five months, from October 2022 to March 2023, all nurses working in the same hospital located in a medium-sized city (with approximately 200.000 inhabitants) in northeastern Italy were asked to take part in the study during routine preventive occupational medicine consultation. The majority of nurses accepted the invitation to participate (acceptance rate = 92%), and written informed consent was collected from each participant. A research assistant measured participants' weight and height for body mass index [BMI] calculation. Subsequently, participants were asked to complete a paper-and-pencil questionnaire and return it in a closed urn to ensure anonymity.

The study was approved by the Ethical Committee of Friuli-Venezia Giulia (ID: 16810) and was conducted in accordance with the principles outlined in the Helsinki Declaration.

### 2.2. Measures

Participants received a booklet divided into two sections. The first section contained the Italian version of the HSE-MS IT [34], a 35-item questionnaire designed to assess exposure to organizational stress factors based on the HSE Management Standards framework [21]. The HSE-MS IT considers a six-month time window prior to measurement and consists of seven scales: Demands (8 items), Control (6 items), Managers' support (5 items), Peer support (4 items), Relationships (4 items), Role (5 items), and Change (3 items). Higher scores on the HSE-MS IT scales indicate a lower risk of stress.

The second section included eight items measuring the prevalence of a set of psychosomatic complaints (palpitations, sleep disorders, depression, irritability, anxiety, physical and mental tiredness, headache, and osteoarticular pain) experienced over the last six months. These complaints are commonly associated with work-related stress [25],[26],[35] and were assessed using a five-point scale (ranging from never to always).

### 2.3. Data analysis

Mean scores and standard deviations were calculated for each of the seven HSE-MS IT scales, and compared with Italian benchmark data [36]. Descriptive statistics were also provided for nurses' psychosomatic complaints. To assess associations between HSE-MS IT scales and psychosomatic complaints, Pearson correlations were calculated between the HSE-MS IT scales and the psychosomatic complaints. Subsequently, hierarchical logistic regressions were conducted with each complaint as outcome variables and the HSE-MS IT scales as predictors, after controlling for gender, age group, and BMI. HSE-MS IT scores below the 20th percentile of the benchmark data were coded as 1 to indicate a high stress risk, while scores above the 20th percentile were coded as 0. Psychosomatic complaints scores were dichotomized to distinguish between nurses reporting low prevalence (1–3, coded as 0) and high prevalence (4–5, coded as 1). This way, Odds Ratio (OR) and their respective 95% Confidence Intervals (95% CI) were calculated for each psychosomatic complaint, adjusting for the effects of gender, age, and BMI. Multicollinearity was assessed prior to data analysis, with a variance inflation factor (VIF) of less than 5 set as the cutoff value. All analyses were conducted using Jamovi software.

## 3. Results

The final sample consisted of 215 nurses, and their demographic characteristics are reported in Table 1.

**Table 1.** Demographic characteristics of the sample.

Age group	Gender	
	M	F
<30	3	22
30–40	7	40
41–50	14	34
51–60	15	74
61–65	1	3
Total	40	173

Note: Two participants did not report their gender

Descriptive statistics of the HSE-MS IT scales are provided in Table 2. Compared to Italian benchmark data, the average scores for Demands, Managers' support, Relationships, and Change fell between the 20th and 50th percentiles (labeled as ‘‘Clear need for improvement”), while scores for the others scales were above the 50th percentile (labeled as ‘‘Good, but need for improvement”).

**Table 2.** Descriptive statistics of the HSE-MS IT scales.

HSE-MS IT scale	*Mean (SD)*	Benchmark comparison
Demands	3.28 (0.60)	<50th percentile
Control	3.55 (0.66)	>50th percentile
Managers' support	3.79 (0.98)	<50th percentile
Peer support	4.13 (0.62)	>50th percentile
Relationships	3.93 (0.75)	<50th percentile
Role	4.52 (0.47)	>50th percentile
Change	3.66 (0.82)	<50th percentile

As shown in Table 3, sleep disorders and tiredness (physical and mental) were the most prevalent complaints, with approximately 40% of the nurses reporting experiencing them often or always. Palpitations, depression and anxiety were instead the least prevalent complaints, with fewer than 20% of nurses reporting a high frequency.

**Table 3.** Prevalence of the psychosomatic complaints.

Psychosomatic symptom	*Mean (SD)*	% of scores ≥ 4
Palpitations	2.23 (1.05)	13.5%
Sleep disorders	3.04 (1.18)	39.8%
Depression	2.38 (1.11)	17.5%
Irritability	2.62 (1.04)	21.9%
Anxiety	2.24 (1.14)	15.8%
Physical and mental tiredness	3.17 (1.07)	43.3%
Headache	2.38 (1.17)	20.3%
Osteoarticular pain	2.49 (1.29)	26.8%

Correlational analyses (Table 4) revealed significant associations between psychosomatic complaints and the organizational stressors measured by the HSE-MS Indicator Tool scales.

**Table 4.** Correlations among HSE-MS IT scales and psychosomatic complaints.

Project	D	C	MS	PS	RE	RO	C
Palpitations	-0.21**	-0.02	-0.03	-0.05	-0.24***	-0.06	-0.14*
Sleep disorders	-0.18**	-0.13	-0.27***	-0.20**	-0.34***	-0.16*	-0.24***
Depression	-0.36***	-0.15*	-0.23***	-0.19**	-0.39***	-0.19**	-0.27***
Irritability	-0.39***	-0.24***	-0.24***	-0.21**	-0.42***	-0.20**	-0.30***
Anxiety	-0.21***	-0.12	-0.19**	-0.16*	-0.32***	-0.29***	-0.23***
Physical and mental tiredness	-0.42***	-0.26***	-0.17*	-0.16*	-0.39***	-0.27***	-0.26***
Headache	-0.09	-0.05	-0.06	-0.16*	-0.14*	-0.09	-0.12
Osteoarticular pain	-0.19**	0.01	-0.08	-0.15*	-0.19**	-0.16*	-0.07

Note: D = Demands, C = Control, MS = Managers' support, PS = Peer support, RE = Relationships, RO = Role, C = Change. *p < 0.05, **p < 0.01, ***p < 0.001.

To provide a more nuanced understanding of the contribution of each organizational stressor to psychosomatic complaints, multiple regression analyses were conducted, controlling for gender, age group, and BMI.

Table 5 presents the ORs between exposure to organizational stressors and psychosomatic complaints. The Relationships factor was associated with an increased risk of experiencing palpitations (3.89 times), irritability (4.85 times), anxiety (3.38 times), physical and mental tiredness (7.09 times), and headache (3.04 times). Additionally, Demands increased the risk of experiencing irritability (2.51 times) and physical and mental tiredness (2.65 times), while Managers' support was a significant risk factor for depression (2.74 times). No significant associations were found between organizational stress factors and sleep disorders or osteoarticular pain.

**Table 5.** Associations between organizational stressors and psychosomatic complaints.

Predictors	Palpitations	Sleep disorders	Depression	Irritability	Anxiety	Physical and mental tiredness	Headache	Osteoarticular pain

	*OR* (95% *CI*)	*OR* (95% *CI*)	*OR* (95% *CI*)	*OR* (95% *CI*)	*OR* (95% *CI*)	*OR* (95% *CI*)	*OR* (95% *CI*)	*OR* (95% *CI*)
Age group	0.67* (0.45–0.98)	1.34 (1.00–1.78)	0.87 (0.61–1.25)	0.76 (0.54–1.06)	0.87 (0.60–1.27)	1.16 (0.85–1.56)	0.85 (0.60–1.20)	2.35*** (1.56–3.55)
Gender (female)	5.45* (1.11–26.66)	2.18 (0.95–5.02)	1.74 (0.56–5.34)	2.31 (0.73–7.31)	2.34 (0.68–8.09)	2.63* (1.05–6.59)	5.65* (1.50–21.20)	4.16* (1.30–13.31)
BMI	2.88* (1.13–7.34	1.52 (0.80–2.87)	1.84 (0.82–4.13)	0.84 (0.38–1.85)	2.21 (0.95–5.14)	2.57* (1.30–5.09)	3.63** (1.64–8.04)	2.24* (1.09–4.63)
Demands	0.66 (0.24–1.82)	1.37 (0.69–2.71)	2.01 (0.87–4.64)	2.51* (1.14–5.52)	1.36 (0.57–3.26)	2.65** (1.29–5.42)	0.75 (0.32–1.75)	1.92 (0.88–4.17)
Control	0.75 (0.18–3.04)	0.94 (0.37–2.38)	0.49 (0.13–1.78)	0.51 (0.16–1.63)	0.50 (0.13–1.92)	2.67 (0.91–7.79)	0.81 (0.25–2.63)	1.35 (0.45–4.00)
Managers' support	1.57 (0.49–5.05)	2.02 (0.87–4.71)	2.74* (1.01–7.44)	1.55 (0.57–4.17)	1.68 (0.58–4.83)	0.52 (0.19–1.42)	1.19 (0.43–3.33)	0.62 (0.22–1.76)
Peer support	0.48 (0.05–4.93)	2.07 (0.42–10.12)	1.24 (0.24–6.43)	0.29 (0.04–1.86)	0.50 (0.08–3.16)	0.61 (0.10–3.60)	1.23 (0.24–6.26)	0.35 (0.06–2.22)
Relationships	3.89* (1.10–13.79)	1.58 (0.59–4.27)	1.36 (0.43–4.29)	4.85** (1.66–14.19)	3.38* (1.11–10.29)	7.09** (1.93–26.03)	3.04* (1.02–9.08)	1.53 (0.49–4.80)
Role	0.49 (0.09–2.60)	0.74 (0.26–2.06)	2.09 (0.70–6.31)	2.62 (0.88–7.78)	2.72 (0.89–8.31)	2.22 (0.68–7.31)	1.06 (0.33–3.42)	1.54 (0.50–4.68)
Change	0.70 (0.20–2.45)	1.36 (0.80–2.87)	0.83 (0.29–2.39)	0.91 (0.34–2.47)	1.02 (0.34–3.02)	1.33 (0.51–3.49)	1.04 (0.37–2.92)	0.92 (0.32–2.67)

## 4. Conclusions

The recognition of the negative effects of organizational stressors, both on nurses' health and on the quality of patient care and organizational effectiveness, underscores the relevance of investigating their implications comprehensively. Addressing these challenges necessitates a nuanced understanding of the specific organizational stressors prevalent in nursing contexts and their relationship with health outcomes.

We investigated the associations between exposure to the seven organizational stressors of the HSE Management Standards framework, and a range of psychosomatic complaints among nurses in a medium-sized city hospital in northeastern Italy. Consistent with previous research, our results revealed significant associations between various organizational stressors and the prevalence of psychosomatic complaints [26],[27]. Specifically, the Relationships factor emerged as a significant predictor of several psychosomatic complaints, including palpitations, irritability, anxiety, physical and mental tiredness, and headache. This result underscores the critical role of interpersonal relationships within the workplace in influencing nurses' mental and physical health outcomes [10],[37],[38]. Additionally, Demands and Managers' support were identified as significant predictors of specific psychosomatic complaints (irritability and physical and mental tiredness, and depression, respectively), further highlighting the multifaceted nature of organizational stress and the need for targeted interventions to address diverse stressors in the nursing profession.

These results are in line with the broader literature on organizational stress and its impact on healthcare professionals' well-being. For example, studies among other healthcare professionals, such as radiologists, have similarly highlighted the detrimental effects of organizational stressors on mental and physical health outcomes [39],[40]. By acknowledging the broader literature on organizational stress and its impact on healthcare professionals, we gain a deeper understanding of the complex dynamics at play in healthcare settings. Moving forward, interventions aimed at mitigating organizational stressors should consider the unique challenges faced by different healthcare professionals and prioritize the creation of supportive work environments that promote well-being and optimal patient care delivery.

The strengths of this study include the use of a well-established measurement tool, the HSE-MS IT, to assess organizational stressors, and a comprehensive assessment of a spectrum of psychosomatic complaints commonly associated with work-related stress. Furthermore, the large sample size and high participation rate enhance the reliability of our findings.

However, some limitations need to be acknowledged. Initially, the cross-sectional design precludes causal inference, and longitudinal studies are needed to establish temporal relationships between organizational stressors and psychosomatic complaints. Additionally, the use of self-report measures introduces the potential for response bias, and future research could benefit from incorporating objective measures or observational data to corroborate self-reported findings. Furthermore, it is plausible that the COVID-19 pandemic exacerbated stress among nurses. Although data collection occurred after the onset of the pandemic, nurses directly experienced its effects, which included an unforeseen surge in workload and uncertainty within what they perceived as a hostile environment [41]–[43]. Consequently, the obtained results may have been influenced by pandemic-related fatigue, potentially exacerbating perceptions of workload and straining professional relationships. Another limitation of this study is the potential influence of social desirability bias, wherein participants may have responded in a manner they deemed socially acceptable than providing honest or accurate responses, in order to portray themselves and their organization in a favorable light. This bias could be particularly relevant in the healthcare sector, given the numerous challenges and criticisms faced by healthcare professionals during the COVID-19 pandemic [44]. Future research could employ alternative methodologies, such as mixed methods incorporating both questionnaires and interviews or focus groups, to mitigate this bias and further investigate the impact of organizational stressors on nurses' well-being.

Despite these limitations, our study provides valuable insights into the complex interplay between organizational stressors and nurses' health outcomes. By identifying specific stressors that contribute to psychosomatic complaints, healthcare organizations can implement targeted interventions to mitigate these stressors and promote a healthier work environment for nurses. In their systematic review, Cohen and colleagues [45] found that the majority of interventions aimed at improving the well-being of healthcare workers employed both individual and organizational approaches. At the individual level, interventions were predominantly focused on secondary prevention strategies, such as stress management techniques including mindfulness-based practices, meditation, yoga, acupuncture, and fostering a positive mindset. Organizational-level interventions encompassed measures to alleviate workload, encourage job crafting, and establish peer support networks. Notably, the authors reported that most studies documented positive outcomes, including enhancements in well-being, increased work engagement, and reductions in burnout, perceived stress, anxiety, and depression symptoms.

In conclusion, our study underscores the importance of addressing organizational stressors in nursing practice, including those related to interpersonal relationships within the workplace. The significant associations between the Relationships factor and various psychosomatic complaints highlight the critical role of fostering positive social interactions in promoting nurses' mental and physical well-being. Thus, by prioritizing the creation of supportive work environments and implementing evidence-based interventions, healthcare organizations can foster a culture of well-being among nurses, ultimately enhancing the quality and safety of healthcare delivery.

## Use of AI tools declaration

The authors declare no Artificial Intelligence (AI) tools have been used in the creation of this article.
